# The off-hour effect on trauma patients requiring subspecialty intervention at a community hospital in Japan: a retrospective cohort study

**DOI:** 10.1186/s13049-015-0095-1

**Published:** 2015-02-10

**Authors:** Yuko Ono, Tokiya Ishida, Yudai Iwasaki, Yutaka Kawakami, Ryota Inokuchi, Choichiro Tase, Kazuaki Shinohara

**Affiliations:** Emergency and Critical Care Medical Center, Ohta General Hospital Foundation, Ohta Nishinouchi Hospital, 2-5-20 Nishinouchi, 963-8558 Koriyama, Fukushima Japan; Emergency and Critical Care Medical Center, Fukushima Medical University Hospital, 1 Hikarigaoka, 960-1295 Koriyama, Fukushima Japan; Department of Emergency and Critical Care Medicine, The University of Tokyo Hospital, 7-3-1 Hongo, Bunkyo-ku, 113-8655 Tokyo Japan

**Keywords:** Complications, Emergency surgery, Night presentation, Preoperative period, Transarterial embolization, Unexpected trauma death, Weekend presentation

## Abstract

**Background:**

Because most community hospitals in Japan do not maintain 24-h availability of in-house anesthesiologists, surgeons, and interventional radiologists, staffing dramatically declines during off hours. It is unclear whether, in such under-resourced hospitals, trauma patients presenting during off hours and requiring subspecialty intervention have worse outcomes than those who present during business hours.

**Methods:**

This was a retrospective cohort study at a community hospital in Japan. Participants were all injured patients requiring emergency trauma surgery or transarterial embolization who presented from January 2002 to December 2013. We investigated whether outcomes of these patients differed between business hours (8:01 AM to 6:00 PM weekdays) and off hours (6:01 PM to 8:00 AM weekdays plus all weekend hours). The primary outcome measure was mortality rate, and the secondary outcome measures were duration of emergency room (ER) stay; unexpected death (death/probability of survival > 0.5); and adverse events occurring in the ER. We adjusted for potential confounders of age, sex, Injury Severity Score (ISS), Revised Trauma Score, presentation phase (2002–2005, 2006–2009, and 2010–2013), Charlson Comorbidity Index, and injury type (blunt or penetrating) using logistic regression models.

**Results:**

Of the 805 patients included, 379 (47.1%) presented during business hours and 426 (52.9%) during off hours. Off-hours presentation was associated with longer ER stays for patients with systolic blood pressure < 90 mmHg on admission (*p* = 0.021), ISS >15 (*p =* 0.047), and pelvic fracture requiring transarterial embolization (*p* < 0.001). Off-hours presentation was also associated with increased risk of adverse events in the ER (odds ratio [OR] 1.7, 95% confidence interval [CI] 1.1–2.7, *p* = 0.020). After adjustment for confounders, an increased risk of adverse events (OR 1.6, 95% CI 1.1–2.7, *p* = 0.049) persisted, but no differences were detected in mortality (*p* = 0.80) and unexpected death (*p* = 0.44) between off hours and business hours.

**Conclusions:**

At a community hospital in Japan, presentation during off hours was associated with a longer ER stay for severely injured patients and increased risk of adverse events in the ER. However, these disadvantages did not impact mortality or unexpected outcome.

## Background

At most medical institutions, staffing levels dramatically decrease during off hours; i.e., nights and weekends. At such times, experienced doctors in supervisory roles and consultants in subspecialties are less available [1], and staff performance can be impaired because of fatigue and disrupted circadian rhythms [2]; consequently, more medical errors [3,4] and complications [5] tend to occur. Off-hour presentation is therefore known to be a risk factor for patients presenting with unplanned critical conditions requiring rapid diagnosis and aggressive intervention, including cardiac arrest [6], myocardial infarction [7], stroke [1], ruptured aortic aneurysm [8], acute epiglottis [8], and pulmonary embolism [8]. This phenomenon is termed the *off-hour effect*. Previous reports have shown that, if patients are treated at a level I trauma center in a mature trauma-care system, mortality rates of those presenting during off hours are not worse than those of patients presenting during business hours [9-14]. A level I trauma center [15] has the highest concentration of medical resources, including 24-hour availability of in-house surgeons, anesthesiologists, and interventional radiologists.

Unfortunately, such specialized trauma care has not yet been implemented everywhere in Japan. Medical staff shortage is one of the most serious problems, especially in the provinces [16,17]. For example, our institution, a community hospital in Japan, requires only one attending emergency room (ER) physician and one resident to be available to treat injured patients during off hours, with surgeons, anesthesiologists, and interventional radiologists more likely to be on call for emergency trauma surgery or transarterial embolization (TAE). Similar to our hospital, most Japanese community hospitals do not comply with American College of Surgeons standards for a level I [15], or even a level II, trauma center [15]. We are not aware of any studies that have examined the off-hour effect on trauma care and outcomes in such settings. Therefore, we conducted this study of patients in a representative under-resourced hospital in a developing trauma care system to determine whether the care and outcomes of injured patients, especially those requiring subspecialty intervention, differ significantly between off hours and business hours.

## Methods

### Study design and setting

This was a retrospective cohort study conducted at a community hospital in a provincial Japanese city approximately 200 km north of Tokyo. The hospital serves as a teaching facility and a referral medical center that receives over 1,400 trauma patients per year, with injuries of varying severity, from areas within a 50-km radius. Two or three attending ER physicians (postgraduate year >3) and two or three residents (post graduate year 1 or 2) take part in the initial management of trauma patients during business hours, and one attending ER physician and one resident are present during off hours. There is 24-h staffing with ER physician(s), resident(s), and operating room (OR) nurses, but surgeons, anesthesiologists, and interventional radiologists are not available 24 h per day. If injured patients require emergency trauma surgery or TAE, staff members respond immediately from in-house during business hours but from outside the hospital during off hours. In most cases, the response time (time elapsed from call to presence at the ER) during off hours is 30 min or less.

### Participants and data sources

After approval by the institutional review boards at the authors’ institutions, we reviewed the records of all injured patients requiring open reduction with internal fixation (ORIF) for open fractures of extremities or laparotomy, craniotomy, or thoracotomy who were brought directly from the ER to the OR and all patients with pelvic fractures requiring TAE who were brought directly from the ER to the catheterization laboratory from January 1, 2002, to December 31, 2013. Data were collected from pre-hospital records, ER records, medical records, nursing records, anesthesia records, and an electronic database that included adverse events occurring in the ER. At our hospital we use a common form for ER records that includes arrival vital signs, time course, past medical history, a detailed history of the present condition, physical examination, final diagnosis, and any adverse events. All doctors who participate in management of the trauma are obliged to complete the form immediately, and a trauma director at our hospital (author KS) checks all medical records to verify the completeness and reliability of data. Our department maintains a rigorous peer-review process to ensure the quality of our ER practice. Any adverse events occurring in our ER are peer reviewed, confirmed by experienced ER physicians, and recorded to the electronic database without delay.

### Exposures and outcome measurements

The primary exposure was presentation during off hours. We compared clinical characteristics, trauma care, and outcomes of injured patients requiring subspecialty interventions who presented during off hours with those who presented during business hours. Business hours were defined as the period from 8:01 AM to 6:00 PM weekdays, and off hours were defined as the period from 6:01 PM to 8:00 AM weekdays plus all weekend.

Clinical characteristics included age; sex; injury severity as represented by Injury Severity Score (ISS) [18,19], Revised Trauma Score (RTS) [20,21], and probability of survival (Ps) based on trauma and injury-severity scores [22-24]; Glasgow Coma Scale and vital signs (systolic blood pressure [SBP], heart rate, and respiratory rate) measured immediately after admission to the ER; American Society of Anesthesiologists Physical Status (ASA-PS); Charlson Comorbidity Index (CCI) [25,26]; and injury type (blunt or penetrating). The CCI [25,26] is a weighted index of the number of serious comorbidities on a scale from 0 (no comorbid disease) to 8 (serious comorbid disease). All data for clinical characteristics except CCI were recorded prospectively. ISS, RTS, and Ps were scored immediately by author KS. ISS was scored based on anatomical information obtained by physical examination, x-ray, computed tomography (CT), and operative findings. RTS was scored based on vital signs measured immediately after ER admission. ASA-PS was scored by attending anesthesiologists, and CCI was scored retrospectively by author YO.

Our study period was quite long, and during this time a standardized trauma education program (Japan Advanced Trauma Evaluation and Care™) was introduced throughout Japan, including at our facility. Because of the report by Hondo et al. [27] that introduction of this trauma education program could affect trauma care and outcomes, we separated our sample into their three phases (2002–2005, 2006–2009, and 2010–2013) [27] and considered phase as a possible confounder. Our hospital resources, however, including staffing and the on-call system during off hours, remained relatively unchanged during the study period.

Trauma care parameters were pre-hospital time (time from emergency call to ER arrival), ER stay time (time from ER arrival to OR), and total time to OR (time from emergency call to OR). We adopted preoperative elapsed time as a care parameter because early operative control of hemorrhage is vital in injured patients [28] and is considered by many previous studies to be an important parameter of trauma care [29-32]. OR arrival was defined as the anesthesia start time documented in anesthesia records. TAE was defined as the time of arrival at the catheterization laboratory documented in nursing records. Patients were subcategorized into shock (SBP < 90 mmHg) on arrival to the ER, penetrating injury, and severe injury (ISS > 15) and into each subspecialty intervention for subgroup analysis.

The primary outcome measure was mortality rate. Secondary outcome measures were unexpected trauma death (Death/Ps > 0.5), good recovery, and adverse events occurring in the ER. Good recovery was defined as either discharge to home without home nursing care, or transfer to an inpatient rehabilitation facility. Adverse events occurring in the ER were first extracted from our electronic database. After masking information about presentation time (business hours or off hours) to minimize bias and increase inter-rater reliability, these adverse events were verified independently by another ER physician according to the following definitions: *Missed major injury,* any injury missed in the ER that required further invasive treatment or change in treatment plan; *device infection,* any infection, confirmed by inflammation of insertion site, fever, or catheter or tube culture if possible, related to an indwelling device inserted in the ER and requiring removal; *device malposition,* any misplacement of a tube (other than an endotracheal tube), drain, or catheter inserted in the ER confirmed by CT or x-ray; *endotracheal intubation complications*, adverse events associated with inducting-agent administration, laryngoscopy, or tube placement (e.g., upper airway trauma, hypoxemia, or dysrhythmia) in the ER; *delay in intervention,* failure of non-operative management because of hemorrhage or deterioration of vital signs; *postoperative bleeding requiring reoperation; and iatrogenic injury*, any treatment-associated injury occurring in the ER (e.g., pneumothorax caused by insertion of a central venous catheter).

We also evaluated whether distributions of time and cause of death differed between business and off-hour presentation. Time-of-death distribution was defined as the interval from ER arrival to the occurrence of death. Cause of death was extracted from the death certificate.

### Power analysis

Power analysis was performed using G*Power 3 for Windows (Heinrich Heine University, Düsseldorf, Germany). Assuming an 8.0% death rate for injured patients who require subspecialty intervention and are admitted during business hours (based on our 2002–2005 pilot data, and consistent with data from another report [13]), a sample size of 288 patients per group provides 80% statistical power to detect a 5% mortality difference for off-hours presentation at a two-sided significance level of *p* < 0.05.

### Statistical analysis

To evaluate differences in clinical characteristics between off hours and business hours, we used the Mann–Whitney *U*-test to compare continuous variables and Fisher’s exact test for categorical variables. We next determined the outcome differences, including mortality, adverse events, good recovery, and unexpected trauma death, between business-hours and off-hours presentation using both unadjusted and adjusted logistic regression models. We adjusted for potential confounders shown to be associated with outcomes of trauma patients, including age [33,34], sex [35-37], ISS [18,19], RTS [20,21], CCI [38,39], presentation phase (2002–2005, 2006–2009, or 2010–2013) [27], and injury type (blunt or penetrating) [23,24]. We used a variance-inflation factor to detect multicollinearity and used the Hosmer–Lemeshow test to verify model fit. A *p* value < 0.05 was considered statistically significant. All statistical analyses except power analysis were performed using IBM SPSS Statistics for Windows, Version 21.0 (IBM Corp., Armonk, NY, USA).

## Results

During the study period, 15,480 injured patients were brought to the ER, of whom 805 (5.2%; mean age 47.2 ± 22.2 years; 71.6% male; ISS 18.6 ± 14.6, 13.3% penetrating injuries) required emergency trauma surgery or TAE. There were no missing data, and no patients were excluded from this analysis. The distribution of emergency surgeries was 507 ORIFs for open fractures (63.0%); 144 TAEs for pelvic fractures (17.9%); 118 laparotomies (14.7%); 27 craniotomies (3.4%); and nine thoracotomies (1.1%). Of these 805 patients, 379 (47.1%) were admitted to the hospital during business hours and 426 (52.9%) during off hours. Table 1 presents comparisons of clinical characteristics by time of presentation. Patients admitted during off hours were significantly younger (*p* = 0.0030) and had a significantly higher ISS (*p* < 0.001) and lower RTS (*p* = 0.042) than those admitted during business hours. No differences were detected in other clinical characteristics or in distribution of subspecialty intervention by time of presentation.

**Table 1:** **Clinical characteristics of injured patients: business hours** ^**1**^ **vs. off hours** ^**2**^

**Characteristic**	**All (n = 805)**	**Business hours (n = 379)**	**Off hours (n = 426)**	***p*** **value**
**Age (years)**	47.2 ± 22.2	49.6 ± 22.7	45.1 ± 21.6	0.0030
**Male, n (%)**	576 (71.6)	270 (71.2)	306 (71.8)	0.88
**ISS**	18.6 ± 14.6	17.1 ± 14.6	19.9 ± 14.4	<0.001
**RTS**	7.3 ± 1.2	7.3 ± 1.2	7.2 ± 1.2	0.042
**Ps**	0.88 ± 0.23	0.88 ± 0.24	0.88 ± 0.23	0.315
**Glasgow Coma Scale**	13.5 ± 3.0	13.6 ± 2.9	13.4 ± 3.1	0.054
**SBP (mmHg)**	123.9 ± 58.1	124.2 ± 33.2	123.6 ± 73.5	0.064
**Heart rate (beats per min)**	89.8 ± 23.0	89.0 ± 24.2	90.6 ± 21.9	0.092
**Respiratory rate (breaths per min)**	20.0 ± 7.9	19.8 ± 8.0	20.3 ± 7.7	0.253
**ASA-PS**	2.7 ± 1.0E	2.7 ± 0.9E	2.8 ± 1.0E	0.30
**CCI**	0.37 ± 0.82	0.43 ± 0.91	0.33 ± 0.73	0.48
**Penetrating injury, n (%)**	107 (13.3)	59 (15.6)	48 (11.3)	0.078
**ORIF, n (%)**	507 (63.0)	251 (66.2)	256 (60.1)	0.079
**TAE, n (%)**	144 (17.9)	63 (16.6)	81 (19.0)	0.41
**Laparotomy, n (%)**	118 (14.7)	48 (12.7)	70 (16.4)	0.14
**Craniotomy, n (%)**	27 (3.4)	11 (2.9)	16 (3.8)	0.56
**Thoracotomy, n (%)**	9 (1.1)	6 (1.6)	3 (0.7)	0.32
**Presentation 2002–2005**	314 (39.0)	148 (39.1)	166 (39.0)	1.00
**Presentation 2006–2009**	233 (28.9)	98 (25.9)	135 (31.7)	0.074
**Presentation 2010–2013**	258 (32.0)	133 (35.1)	125 (29.3)	0.083

^1^8:01 AM to 6:00 PM weekdays.

^2^6:01 PM to 8:00 AM weekdays plus all weekend hours.

Data expressed as mean ± standard deviation or n (%).

ASA-PS, American Society of Anesthesiologists Physical Status; CCI, Charlson Comorbidity Index; ISS, Injury Severity Score; ORIF, open reduction with internal fixation; Ps, probability of survival; RTS, Revised Trauma Score; SBP, systolic blood pressure; TAE, transarterial embolization.

Table 2 presents comparisons of care and outcomes between business hours and off hours. Off-hours presentation was associated with increased risk of adverse events in the ER (odds ratio [OR] 1.7, 95% confidence interval [CI] 1.1–2.7, *p* = 0.020). ER stays were significantly longer during off hours than during business hours in the shock group (*p =* 0.021) and ISS >15 group (*p* = 0.047). Pre-hospital time and rates of mortality, unexpected trauma death, good recovery, and preventable complications were not associated with ER admission period in any group.

**Table 2:** **Care and outcome parameters of injured patients: business hours** ^**1**^ **vs. off hours** ^**2**^

	**All**		**Shock (SBP < 90 mmHg)**		**Penetrating injury**		**ISS > 15**	
**Parameter**	**Business hours (n = 379)**	**Off hours (n = 426)**	**Business hours (n = 59)**	**Off hours (n = 81)**	**Business hours (n = 59)**	**Off hours (n = 48)**	**Business hours (n = 152)**	**Off hours (n = 218)**
**Prehospital time (min)**	50.4 ± 25.0	50.7 ± 25.7	52.8 ± 23.2	51.3 ± 27.4	41.2 ± 20.6	43.9 ± 22.1	56.2 ± 27.3	53.0 ± 26.7
**ER stay time (min)**	143.1 ± 80.2	144.9 ± 67.7	110.9 ± 69.4	133.1 ± 72.1*	132.4 ± 85.9	115.1 ± 48.5	140.7 ± 85.7	147.4 ± 69.0*
**Total time to OR (min)**	193.5 ± 83.8	195.6 ± 69.1	163.7 ± 71.7	184.4 ± 73.7*	173.5 ± 88.8	159.0 ± 54.6	196.9 ± 90.8	200.4 ± 69.9
**Mortality, n (%)**	32 (8.4)	34 (8.0)	18 (30.5)	21 (25.9)	0 (0)	0 (0)	31 (20.4)	32 (14.7)
**Unexpected trauma death** ^**3**^ **(%)**	12/342 (3.5)	18/391 (4.6)	4/ 32 (12.5)	7/54 (13.0)	0/0 (0)	0/0 (0)	11/115 (9.6)	16/183 (8.7)
**Good recovery, n (%)**	318 (83.9)	361 (84.7)	35 (59.3)	52 (64.2)	54 (91.5)	44 (91.7)	110 (72.4)	169 (77.5)
**Adverse events in the ER, n (%)**	33 (8.7)	60 (14.1)*	10 (16.9)	17 (21.0)	1 (1.7)	1 (2.1)	24 (15.8)	46 (21.1)

^1^8:01 AM to 6:00 PM weekdays.

^2^6:01 PM to 8:00 AM weekdays plus all weekend hours.

^3^Death/Ps>0.5.

Data expressed as mean ± standard deviation unless otherwise indicated. *p < 0.05. ER, emergency room; ISS, Injury Severity Score; OR, operating room; Ps, probability of survival; RTS, Revised Trauma Score; SBP, systolic blood pressure.

Table 3 presents comparisons of patient characteristics; Table 4 presents comparisons of care and outcomes between business hours and off hours by subspecialty intervention. Off-hours presentation was associated with increased risk of adverse events in patients undergoing ORIF (OR 2.5, 95%CI 1.2–5.1, *p* = 0.016). Patients with pelvic fracture requiring TAE had longer ER stay times (*p* < 0.001) and total time to catheterization laboratory (*p* < 0.001) during off hours than during business hours, but there were no significant differences in clinical characteristics or other outcome parameters between off-hours and business-hours presentation. The mortality rate of patients undergoing craniotomy was significantly lower during off hours (*p* = 0.002) than during business hours, but other clinical characteristics and care and outcome parameters did not differ by period of ER admission.

**Table 3:** **Clinical characteristics of injured patients: business hours** ^**1**^ **versus off hours** ^**2**^ **by subspecialty intervention**

	**ORIF**		**TAE**		**Laparotomy**		**Craniotomy**		**Thoracotomy**	
**Characteristic**	**Business hours (n = 251)**	**Off hours (n = 256)**	**Business hours (n = 63)**	**Off hours (n = 81)**	**Business hours (n = 48)**	**Off hours (n = 70)**	**Business hours (n = 11)**	**Off hours (n = 16)**	**Business hours (n = 6)**	**Off hours (n = 3)**
**Age (years)**	46.8 ± 22.8	42.7 ± 21.1*	56.9 ± 22.6	53.4 ± 23.0	53.8 ± 21.6	44.9 ± 19.8*	47.6 ± 17.6	42.5 ± 20.7	60.5 ± 11.0	44.3 ± 22.0
**Males, n (%)**	184 (73.3)	191 (74.6)	41 (65.1)	49 (60.5)	31 (64.6)	49 (70.0)	8 (72.7)	14 (87.5)	6 (100.0)	3 (100.0)
**ISS**	11.0 ± 8.6	13.0 ± 10.5*	32.9 ± 17.0	34.0 ± 13.4	23.8 ± 15.8	26.2 ± 12.2	27.4 ± 10.4	29.2 ± 12.9	35.0 ± 12.2	33.3 ± 15.0
**RTS**	7.6 ± 0.8	7.6 ± 0.6	6.8 ± 1.7	6.5 ± 1.8	7.0 ± 1.5	6.9 ± 1.4	6.1 ± 1.4	6.4 ± 1.0	5.8 ± 2.1	7.3 ± 0.5
**Ps**	0.96 ± 0.11	0.96 ± 0.13	0.70 ± 0.34	0.71 ± 0.34	0.80 ± 0.31	0.85 ± 0.22	0.66 ± 0.31	0.79 ± 0.22	0.60 ± 0.44	0.72 ± 0.24
**ASA-PS**	2.3 ± 0.7	2.3 ± 0.7	3.6 ± 0.8	3.7 ± 0.8	3.3 ± 0.8	3.2 ± 0.9	3.3 ± 1.0	3.3 ± 0.7	4.2 ± 0.8	4.3 ± 1.2
**CCI**	0.36 ± 0.9	0.28 ± 0.7	0.46 ± 0.69	0.56 ± 0.96	0.73 ± 1.2	0.21 ± 0.59***	0.36 ± 0.92	0.56 ± 0.89	0.50 ± 0.84	0.0 ± 0.0
**Penetrating injury, n (%)**	51 (20.3)	40 (15.6)	2 (3.2)	1 (1.2)	4 (8.3)	7 (10.0)	1 (9.1)	0 (0.0)	1 (16.7)	0 (0.0)

^1^8:01 AM to 6:00 PM weekdays.

^2^6:01 PM to 8:00 AM weekdays plus all weekend hours.

Data expressed as mean ± standard deviation unless otherwise indicated. *p < 0.05, ***p < 0.001. ASA-PS, American Society of Anesthesiologists Physical Status; CCI, Charlson comorbidity index; ISS, Injury Severity Score; ORIF, open reduction with internal fixation; Ps, Probability of survival; RTS, Revised Trauma Score; TAE, transarterial embolization.

**Table 4:** **Care and outcome parameters of injured patients by subspecialty intervention: business hours** ^**1**^ **versus off hours** ^**2**^

**Parameter**	**ORIF**		**TAE**		**Laparotomy**		**Craniotomy**		**Thoracotomy**	
	**Business hours (n = 251)**	**Off hours (n = 256)**	**Business hours (n = 63)**	**Off hours (n = 81)**	**Business hours (n = 48)**	**Off hours (n = 70)**	**Business hours (n = 11)**	**Off hours (n = 16)**	**Business hours (n = 6)**	**Off hours (n = 3)**
**Prehospital time (min)**	49.1 ± 24.5	48.5 ± 23.8	54.9 ± 28.5	58.1 ± 28.1	53.9 ± 23.5	52.3 ± 28.7	38.1 ± 20.4	45.4 ± 22.0	49.0 ± 23.0	35.3 ± 10.1
**ER stay (min)**	156.5 ± 82.7	153.8 ± 71.0	108.0 ± 66.4	131.6 ± 61.3***	127.4 ± 71.6	126.3 ± 60.1	142.4 ± 63.0	149.3 ± 54.7	81.5 ± 43.3	156.3 ± 56.3
**Total time to OR (min)**	205.6 ± 87.3	202.3 ± 73.0	162.9 ± 73.1	189.7 ± 63.1***	181.3 ± 70.7	178.5 ± 63.1	180.5 ± 60.1	194.7 ± 48.9	130.5 ± 61.1	191.7 ± 50.0
**Mortality, n (%)**	2 (0.80)	2 (0.78)	14 (22.2)	15 (18.5)	6 (12.5)	15 (21.4)	6 (54.5)	0 (0.0)**	4 (66.7)	2 (66.7)
**Unexpected trauma death, n (%)**	1/247 (0.40)	1/249 (0.40)	4/45 (8.9)	5/63 (7.9)	2/40 (5.0)	10/62 (16.1)	2/6 (33.3)	0/14 (0.0)	3/4 (75.0)	2/3 (66.7)
**Good recovery, n (%)**	232 (92.4)	237 (92.6)	45 (71.4)	57 (70.4)	35 (72.9)	53 (75.7)	5 (45.5)	13 (81.3)	1 (16.7)	1 (33.3)
**Adverse events in the ER, n (%)**	11 (4.4)	26 (10.2)*	14 (22.2)	19 (23.5)	5 (10.4)	13 (18.6)	2 (18.2)	1 (6.3)	1 (16.7)	1 (33.3)

^1^8:01 AM to 6:00 PM weekdays.

^2^6:01 PM to 8:00 AM weekdays plus all weekend hours.

Data expressed as mean ± standard deviation or n (%). *p < 0.05; **p < 0.01; ***p < 0.001.

ASA-PS, American Society of Anesthesiologists Physical Status; CCI, Charlson Comorbidity Index; ER, emergency room; ISS, Injury Severity Score; OR, operating room; ORIF, open reduction with internal fixation; Ps, Probability of survival; RTS, Revised Trauma Score; TAE, transarterial embolization.

Table 5 presents detailed distributions of preventable complications by ER admission period. *Missed major injury*, which included liver injury, hemothorax; perforation of the gut, diaphragm, and urinary bladder; extremity fracture, traumatic aortic dissection, brain contusion, and acute epidural hematoma, was more likely to occur during off hours (OR 2.5, 95% CI 1.1–5.7, *p =* 0.025). *Endotracheal-intubation complications* included cardiac arrest immediately after endotracheal intubation attempts, dysrhythmia, desaturation (percutaneous oxygen saturation < 90% during laryngoscopy), regurgitation, upper airway trauma, mainstem bronchus intubation, and vocal cord paralysis. Iatrogenic injury included lung or liver injury caused by insertion of a chest tube, pneumothorax caused by insertion of a central venous catheter, and urethral injury caused by insertion of a Foley catheter.

**Table 5:** **Detailed distribution of adverse events occurring in the ER: business hours** ^**1**^ **vs. off hours** ^**2**^

**Preventable complication**	**All (n = 805)**	**Business hours (n = 379)**	**Off hours (n = 426)**	**OR (95% CI)**	***p*** **-value**
**Missed major injury, n (%)**	30 (3.7)	8 (2.1)	22 (5.2)	2.5 (1.1–5.7)	0.025
**Device infection, n (%)**	13 (1.6)	4 (1.1)	9 (2.1)	2.0 (0.6–6.6)	0.27
**Device malposition, n (%)**	15 (1.9)	5 (1.3)	10 (2.3)	1.8 (0.6–5.3)	0.31
**Endotracheal-intubation complications, n (%)**	16 (2.0)	8 (2.1)	8 (1.9)	0.9 (0.3–2.3)	1.00
**Delayed intervention, n (%)**	9 (1.1)	5 (1.3)	4 (0.9)	0.7 (0.2–2.7)	0.74
**Postoperative bleeding requiring reoperation, n (%)**	6 (0.7)	2 (0.5)	4 (0.9)	1.8 (0.3–9.8)	0.69
**Iatrogenic injury, n (%)**	4 (0.5)	1 (0.3)	3 (0.7)	2.7 (0.3–25.9)	0.63
**Total, n (%)**	93 (11.5)	33 (8.7)	60 (14.1)	1.7 (1.1–2.7)	0.020

^1^8:01 AM to 6:00 PM weekdays.

^2^6:01 PM to 8:00 AM weekdays plus all weekend hours.

CI, confidence interval; ER, emergency room; OR, odds ratio.

Table 6 presents unadjusted and adjusted outcomes between business hours and off hours. In the unadjusted analysis, off-hours presentation was associated with increased risk of adverse events in the ER (OR 1.7, 95% CI 1.1–2.7, *p* = 0.023), but not with mortality (*p* = 0.34); unexpected trauma death (*p =* 0.88); or good recovery (*p =* 0.27). Even after adjusting for possible confounders including age, sex, ISS, RTS, CCI, presentation phase (2002–2005, 2006–2009, and 2010–2013), and injury type using logistic-regression models, there continued to be an increased risk of adverse events associated with off-hours presentation (OR 1.6, 95% CI 1.1–2.7, *p* = 0.049), but no differences were detected in mortality (*p* = 0.80); unexpected trauma death (*p =* 0.44); and good recovery (*p =* 0.80) between off hours and business hours. We did not detect multicollinearity (variance-inflation factor < 2 in each explanatory variable), and the Hosmer–Lemeshow test verified good fit (*p =* 0.70, *p =* 0.15, *p =* 0.56, and *p =* 0.39, respectively) in each model.

**Table 6:** **Unadjusted and adjusted outcomes: business hours** ^**1**^ **vs. off hours** ^**2**^

	**Unadjusted analysis**		**Adjusted analysis**	
**Outcomes**	**Unadjusted OR (95% CI)**	***p*** **-value**	**Adjusted OR (95% CI)**	***p*** **-value**
**Mortality**	0.8 (0.4–1.4)	0.34	0.9 (0.5–1.8)	0.80
**Unexpected trauma death**	1.1 (0.5–2.4)	0.88	1.4 (0.6–3.0)	0.44
**Good recovery**	1.3 (0.8–2.0)	0.27	1.1 (0.7–1.7)	0.80
**Adverse events in the ER**	1.7 (1.1–2.7)	0.023	1.6 (1.1–2.7)	0.049

^1^8:01 AM to 6:00 PM weekdays.

^2^6:01 PM to 8:00 AM weekdays plus all weekend hours.

CI, confidence interval; ER, emergency room; OR, odds ratio.

**Figure 1:**
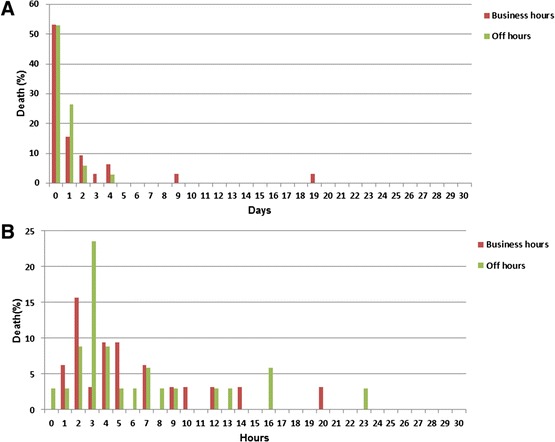
**Time-of-death analysis: business hours** ^**1**^ **vs. off hours** ^**2**^ **. (A)** Deaths within the first 30 days. Deaths usually occurred within the first day for both business-hours (17/32, 53.1%) and off-hours (18/34, 52.9%) presentations. **(B)** Deaths within the first 30 hours. Almost half of deaths occurred within 1–5 hours after presentation for both business-hours (14/32, 43.8%) and off-hours (16/34, 47.1%) presentations. ^1^8:01 AM to 6:00 PM weekdays. ^2^6:01 PM to 8:00 AM weekdays plus all weekend hours.

Figure 1 presents death distribution within the first 30 days and first 30 hours. Deaths occurred predominantly within the first day for both business-hours (17/32, 53.1%) and off-hours (18/34, 52.9%) presentations (Figure 1A), especially within 1–5 hours after ER admission (business hours: 14/32, 43.8%; off hours: 16/34, 47.1%) corresponding to the second peak (deaths within 1–4 hours after trauma) of classic tri-modal trauma-death distribution [40,41] (Figure 1B). The first peak (deaths occurring within the first hour) [40,41] and the third peak (deaths occurring more than 1 week after trauma) [40,41] were absent for both business-hours and off-hours presentations. The main cause of death of injured patients needing subspecialty intervention was hemorrhagic shock (69.7%), followed by brain injury (12.1%) and multiple organ failure (10.6%). Almost all deaths (30/31, 96.8%) within 5 hours after ER admission were caused by hemorrhagic shock. Time- and cause-of-death distributions did not differ between business- and off-hours presentations.

## Discussion

Early operative control of hemorrhage is a key factor in saving the lives of severe trauma patients [28]. Any delay in definitive control of hemorrhage can result in hypovolemic shock and coagulopathy [42], both of which can adversely affect outcomes. Therefore, the care of injured patients requiring emergency trauma surgery is extremely time sensitive. Di Bartolomeo et al. [43] recently studied patients transferred with severe injuries and found that patients who were not brought directly to a level I trauma center were more likely to be affected by the off-hour effect. Di Bartolomeo suggested that the off-hour effect in trauma care could be used as a quality indicator [44]. We tested the hypothesis that the care and outcomes of injured patients requiring subspecialty intervention treated at a community hospital in Japan, which does not comply with American College of Surgeons standards for even a level II trauma center [15], are significantly different between off hours and business hours. We found that 1) Off-hours presentation did not adversely affect survival and unexpected outcome; 2) off-hours presentation was associated with longer ER stays for severely injured patients with SBP < 90 mmHg on admission, ISS >15, or pelvic fracture requiring TAE; 3) adverse events in the ER were more likely to occur during off hours; and 4) time- and cause-of-death distributions between business hours and off hours were similar.

First, we found that off-hours presentation did not adversely affect survival and unexpected outcome in a representative under-resourced hospital. Helling et al. [10] reported that at a level I trauma center there was no significant difference in management or outcome of severely injured patients between those whose arrival time corresponded to the presence of in-house attending trauma surgeons and those who arrived when attending trauma surgeons were generally out of the hospital. Thompson et al. [45] and Barone et al. [46] reported the same results for level II trauma centers. However, while 24-h in-house surgical residents and attending anesthesiologists were available at those institutions, they are not available at our hospital. Staff shortage is one of the most serious healthcare problems in Japan, especially in provincial areas, and most community hospitals share this situation. The present study showed that the response time of surgeons, interventional radiologists, and anesthesiologists from out of hospital does not adversely affect the outcomes of injured patients needing subspecialty intervention as long as they respond in a short time, and as long as initial assessment and care is provided by attending-level ER physician(s) with resident(s). A trained ER staff is able to stabilize the condition of a severely injured patient and maintain it until the arrival of specialists.

Second, we adopted preoperative elapsed time as a care parameter in this study and found that, for patients with shock (SBP < 90 mmHg), severe injury (ISS > 15), and pelvic fracture requiring TAE, ER stay times were significantly longer during off hours than during business hours. Because the survey and resuscitative treatment of such severely injured patients require many hands, declines in staffing during off hours become more apparent. Schwartz et al. [47] also demonstrated that patients with pelvic fractures admitted to the ER at night and on weekends had significantly increased time to TAE and 94% increased risk of mortality compared with those arriving during the daytime and during the week at a level I trauma center. Staffing of emergency medical technicians is constant regardless of time in Japan and is probably why pre-hospital time did not differ between off hours and business hours in any group.

Surprisingly, for patients needing craniotomy, the mortality rate during off hours was better than during business hours even though clinical characteristics and injury severity did not differ significantly in the present study. We could not identify a plausible explanation for this outcome.

Third, we found that adverse events in the ER were more likely to occur during off hours. Among these, risk of missing major injury was particularly increased. There are several possible explanations for this finding; that those who work in hospitals during off hours often have less seniority and experience than those who work during business hours [48,49]; that supervision by experienced doctors is less available [1]; and that the performance of medical staff can be impaired during off hours by fatigue and disrupted circadian rhythms [2]. Our findings suggest that medical providers should be concerned about potential increases in these risks during off hours.

Fourth, we found time- and cause-of-death distributions between business and off hours to be similar. In this study, trauma death usually corresponded to the second peak of the classic tri-modal trauma-death distribution [40,41] regardless of presentation time, and most of those deaths were caused by hemorrhagic shock. Trauma patients with vital organ injury can die before reaching the OR, so our study did not include the first peak [40,41]. De Knegt et al. [50] found that most trauma deaths occurred within the first hour after ER admission (first peak), while the second and third peaks of trauma death were absent in their facility. They suggested that improved trauma care prevented the second peak of trauma death, and that progress in intensive care treatment staved off the third peak [50]. In our study, the second peak of death after trauma was clearly visible during both business hours and off hours, and there was no major improvement associated with presentation time. Conversely, similar time- and cause-of-death distributions between business and off hours suggest that the quality of trauma care does not differ greatly between business hours and off hours.

We also found that trauma patients admitted during off hours were more likely to be younger and to be more severely injured than those admitted during business hours. The same trends have been shown in previous reports [11,47,51]. This could be because younger individuals tend to go out at night or on weekends and may be involved in severe traffic accidents [44] or because nonprofessionals perform high-risk home-maintenance activities on weekends and sustain serious injuries [52].

This report reveals adverse events in detail and differences in care and outcomes between trauma patients requiring subspecialty intervention by time of presentation with no missing data. Most previous investigations of the off-hour effect on trauma patients were based on large trauma databases, but in those studies a considerable amount of data was missing and details of complications and subspecialty care were lacking.

### Limitations

This study has several limitations. First, this is a retrospective cohort study at a single institution. The retrospective and observational nature of this study can increase the risk of bias and introduce possible confounders. We were aware of substantial differences in characteristics between patients who presented during off hours and those who presented during business hours in this study. We rigorously adjusted for known confounders [18-24,27,33-39] to detect differences in outcomes between off hours and business hours, but there is a risk of incomplete adjustment for severity. As with any observational study, there may be other, unmeasured confounders. Our adjustment strategy may have missed subtle but important differences.

Our facility, which is under-resourced and does not have 24-h in-house surgeons, anesthesiologists, and interventional radiologists, is typical of a Japanese trauma-care facility but does not necessarily apply to a well-resourced medical facility in a mature trauma-care system. At our facility, on-call response time is 30 min or less in most cases, but this can vary by facility. Our study also does not necessarily apply to a hospital in which on-call response time is much longer. Nevertheless, we believe our on-call setting is not unusual because every medical facility must ensure that on-call medical staff responds within a *reasonable period of time* [53], which for patients in critical condition is generally considered to be within 30 min [53].

Second, the definition of *adverse events occurring in the ER* in the present study was subjective and not audited by experienced outsiders. We masked presentation time (business hours or off hours), and confirmed adverse events using clear criteria to minimize bias. However, as with any similar study, our strategy may be incomplete. It is possible that there were missed (especially in the case of minor complications), underestimated, or misclassified adverse events. Adverse events may also have been underestimated if they occurred after discharge or transfer to other medical institutions.

Third, this study has a small sample size and may have been underpowered to detect differences in mortality within subgroups i.e., shock, penetrating injury, TAE, laparotomy, craniotomy, and thoracotomy. However, the general trend towards decreased mortality in these subgroups lessens this concern. Further studies with large numbers of patients should be conducted to clarify the off-hour effect on the care and outcomes of injured patients needing subspecialty interventions.

Finally, our surgeons, anesthesiologists, and interventional radiologists are not mandated to remain in-house during off hours, but they often remain on the premises until late at night. We did not take these subspecialists into consideration in this study, but this could have affected care and outcomes.

Despite these limitations, this study reveals the off hour-effect on injured patients requiring subspecialty intervention at a community hospital in Japan. We believe this study represents the current state of trauma care in similar under-resourced hospitals.

## Conclusions

At a community hospital in Japan that does not maintain in-house, 24-h staffing of surgeons, interventional radiologists, and anesthesiologists and that does not comply with American College of Surgeons standards for a level II trauma center, off-hour presentation was associated with longer ER stays for severely injured patients with SBP <90 mmHg on admission, ISS >15, or pelvic fracture requiring TAE. Off-hour presentation was also associated with increased risk of adverse events in the ER, especially missed major injury. Clinicians should be aware that such risks can be increased during off hours; however, these off-hour disadvantages did not impact mortality or unexpected outcome in the present study. This study may represent the current state of trauma care at similar community hospitals in developing trauma-care systems.

## Abbreviations

ASA-PS: American society of anesthesiologists physical status
CCI: Charlson comorbidity index
CI: Confidence interval
ER: Emergency room
ISS: Injury severity score
OR: Operating room
ORIF: Open reduction with internal fixation
OR: Odds ratio
Ps: Probability of survival
RTS: Revised trauma score
SBP: Systolic blood pressure
TAE: Transarterial embolization
